# Pegfilgrastim for primary prophylaxis of febrile neutropenia in multiple myeloma

**DOI:** 10.1007/s00520-021-06266-x

**Published:** 2021-05-14

**Authors:** Claudio Cerchione, Davide Nappi, Giovanni Martinelli

**Affiliations:** 1 Hematology Unit, Istituto Scientifico Romagnolo per lo Studio e la Cura dei Tumori “Dino Amadori” (IRST), IRCCS, Via Piero Maroncelli 40, Meldola, (FC) 47014 Italy; 2 Department of Hematology and Cell Bone Marrow Transplantation (CBMT), Ospedale di Bolzano, Bolzano, Italy

**Keywords:** Pegfilgrastim, Multiple myeloma, G-CSF, Supportive care, Febrile neutropenia

## Abstract

Multiple myeloma (MM) survival rates have been substantially increased thanks to novel agents that have improved survival outcomes and shown better tolerability than treatments of earlier years. These new agents include immunomodulating imide drugs (IMiD) thalidomide and lenalidomide, the proteasome inhibitor bortezomib (PI), recently followed by new generation IMID pomalidomide, monoclonal antibodies daratumumab and elotuzumab, and next generation PI carfilzomib and ixazomib. However, even in this more promising scenario, febrile neutropenia remains a severe side effect of antineoplastic therapies and can lead to a delay and/or dose reduction in subsequent cycles. Supportive care has thus become key in helping patients to obtain the maximum benefit from novel agents. Filgrastim is a human recombinant subcutaneous preparation of G-CSF, largely adopted in hematological supportive care as “on demand” (or secondary) prophylaxis to recovery from neutropenia and its infectious consequences during anti-myeloma treatment. On the contrary, pegfilgrastim is a pegylated long-acting recombinant form of granulocyte colony-stimulating factor (G-CSF) that, given its extended half-life, can be particularly useful when adopted as “primary prophylaxis,” therefore before the onset of neutropenia, along chemotherapy treatment in multiple myeloma patients. There is no direct comparison between the two G-CSF delivery modalities. In this review, we compare data on the two administrations’ modality, highlighting the efficacy of the secondary prophylaxis over multiple myeloma treatment. Advantage of pegfilgrastim could be as follows: the fixed administration rather than multiple injections, reduction in neutropenia and febrile neutropenia rates, and, finally, a cost-effectiveness advantage.

## Introduction

Multiple myeloma (MM) is a clonal plasma cell disorder hallmarked by the uncontrolled proliferation of plasma cell-producing monoclonal proteins that leads to direct and indirect organ damage [1]. Thus, the typical clinical picture of a patient with MM may be represented by renal failure, bone pain and fractures, hypercalcemia, anemia, or other types of cytopenia. MM accounts for about 1% of all cancers and 13% of hematological tumors. It is the second most common hematological malignancy, with an incidence of 6.2 cases per 100,000 individuals [1, 2] with a median range of age at diagnosis of 65–74 years [3]. Although it is not a curable disease, MM survival rates have been substantially improved thanks to the introduction of novel agents such as immunomodulating imide drugs (IMiD) thalidomide and lenalidomide, and the proteasome inhibitor (PI) bortezomib, all of which show improved tolerability and outcome with respect to drugs of earlier years. The therapeutic panorama has further improved following the approval of new generation IMiD pomalidomide, monoclonal antibodies daratumumab and elotuzumab, and next generation PI carfilzomib and ixazomib. [4–6]. However, the advantages of this highly promising therapeutic scenario in a disease that had a poor prognosis until relatively recently must be balanced with the toxicity profile of these compounds. Although these new and newer agents are not “chemotherapy” in the strictest sense of the word, they induce many adverse events that can reduce patient quality of life so severely that treatment discontinuation is needed. In addition to the specific toxicities of each novel and very novel agent currently used to treat MM, mild to severe neutropenia (and associated febrile neutropenia [FN]) is a constant problem [7]. Severe neutropenia leads to a significant risk of harmful, albeit not life-threatening, bacterial, fungal, or mixed infections in hematological malignancies. Given that MM is (considered) a chronic disease of the elderly, with recurrent remission and relapse, infections remain the main cause of death [8]. Preventing neutropenia and febrile neutropenia is thus one of the main aims of ancillary and supportive treatments for patients with MM. Antibiotic prophylaxis (mainly quinolones) is widely used in clinical practice but EORTC guidelines do not recommend its use in hematological and solid tumors as there is a lack of evidence of its efficacy [9, 10]. Thus, the prophylactic use of quinolones should be limited to high-risk patients who are expected to have severe-grade and prolonged neutropenia while undergoing treatment [11]. Filgrastim is a recombinant human G-CSF that stimulates the activation, proliferation, and differentiation of neutrophil progenitor cells. It has been shown to reduce chemotherapy-related neutropenia, lower the incidence of FN, and decrease the need for hospitalization and intravenous antibiotics [12]. Given its short half-life, multiple daily subcutaneous injections of filgrastim are normally required before an optimal neutrophil count is reached. By increasing molecular size through the process of PEGylation, pegfilgrastim has overcome this major pharmacokinetic issue, reducing the plasma clearance rate of filgrastim and prolonging the half-life [13]. The resulting effect is a dramatic reduction in the rate of administration to one subcutaneous injection per cycle of chemotherapy [14].

## Neutropenia and FN in multiple myeloma

Neutropenia is defined by Common Terminology Criteria for Adverse Events (CTCAE) as any decrease in absolute neutrophil count (ANC) below 1500/mm^3^, where moderate and severe neutropenia (grade 3–4) are defined as an ANC < 1000–500/mm^3^ and < 500/mm^3^, respectively (CTCAE version 5.0). Moderate and severe neutropenia, especially if prolonged, can lead to FN, with ANC < 1000/mm^3^ and a single temperature of > 38.3 °C (101°F) or a sustained temperature of ≥ 38 °C (100.4°F) for more than 1 h (CTCAE version 5.0). A direct correlation between a reduced number of neutrophil cells and an increased risk of infection (mainly bacterial and fungal) in MM patients is a fundamental problem in clinical practice [15]. Although neutropenia-related infections can, in themselves, be a severe and potentially life-threatening condition, they may also lead to a delay in the administration of chemotherapy, thus affecting the treatment efficacy and the final clinical outcome. Neutropenia in MM, as in other malignancies, is mainly due to the immunosuppressive and immunomodulating effect of treatment (also novel non-conventional chemotherapeutic compounds) and to the damage caused to mucosal barriers [16]. In addition, MM patients are often immunocompromised by the malfunctioning of immunocompetent cells (B, T, and dendritic cells) caused by clonal plasma cell expansion, constitutional immunosuppression (elderly and frail patients), and intensive and prolonged corticosteroid treatment [8]. In the era of immunomodulating agents, the incidence of infections, especially of bacterial and viral etiology, seems to follow a pattern with two major peaks: at diagnosis/induction and at relapse of disease [17]. A 2015 retrospective evaluation also reported a tenfold higher risk of specific infections, such as pneumonia or septicemia, in the first year after diagnosis of MM, with no major improvements in these rates following the introduction of novel agents [18]. The incidence of neutropenia and FN obviously varies according to the type of treatment regimen adopted. The oldest and/or more aggressive chemotherapeutic agents (or combinations) show the highest rates of severe neutropenia and infections. High-dose melphalan followed by autologous stem cell transplantation (ASCT), the treatment recommended for young and fit patients, has severe neutropenia and infection rates up to 77% and 30%, respectively [19–21]. Other chemotherapeutic combinations used only in highly aggressive disease or in multiple refractory patients, such as VTD-PACE (bortezomib, thalidomide, dexamethasone and cisplatin, doxorubicin, cyclophosphamide, and etoposide) or PACE (cisplatin, doxorubicin, cyclophosphamide, and etoposide) show neutropenia rates of 79% and 83%, respectively, and FN rates of 26% and 33%, respectively [22, 23]. Novel agents such as thalidomide, lenalidomide, and, more recently, pomalidomide (IMiD) are now the backbone of treatment for newly diagnosed and relapsed MM. Neutropenia is one of the most common and predictable adverse events occurring during the use of lenalidomide (along with dexamethasone) and is often managed by treatment discontinuation, dose modulation, and/or G-CSF [24, 25]. The rates of neutropenia and infections from lenalidomide vary according to the dose of dexamethasone and combination compound used, ranging from 32 to 41% and 8 to 22%, respectively [26]. Pomalidomide is a new generation IMiD indicated for the treatment of relapsed/refractory MM (rrMM). Like thalidomide, the most evident adverse event associated with pomalidomide is neutropenia (up to 50% of patients), whereas the rate of associated infections (pneumonia) is lower in proportion (about 13%) [27, 28]. Pomalidomide is often used in heavily pretreated patients who have highly compromised bone marrow function, thus explaining the higher incidence of neutropenia with respect to lenalidomide. It is manageable with dosage modification or treatment withdrawal, and G-CSF support, especially in the first cycles [29]. The first-in-class PI bortezomib, used in association with dexamethasone or other associated molecules (melphalan, lenalidomide), now plays a key role in both newly diagnosed MM (elegible/not eligible) and refractory/relapsed disease [30]. The rates of severe neutropenia and FN are notably lower than those of IMiD, i.e., up to 11% and < 1%, respectively, with very low rates of treatment discontinuation [31]. Carfilzomib, a second-generation PI approved for use in a rrMM setting, induces grade > 3 neutropenia in around 30% of cases, but this rate is probably related to the lenalidomide (and dexamethasone) associated with the regimen [32]. Ixazomib, a newer, orally available PI combined with lenalidomide-dexamethasone, leads to neutropenia rates of about 22%, with a very low incidence of discontinuation for infections [33]. Daratumumab, a newly available anti-CD38 approved for use in rrMM combined with lenalidomide or bortezomib, shows neutropenia rates of about 52% and 12.8%, respectively [34, 35]. Of note, lenalidomide is the leading cause of neutropenia when comparing two regimens containing or not this IMiD. Finally, elotuzumab, a new generation monoclonal antibody directed against SLAMF7 protein, when administered together with lenalidomide for refractory/relapsed MM, has been shown to induce grade > 3 neutropenia rates of 35.5% [36]. Among the drugs approved for use in MM, all IMID, bortezomib and daratumumab, come with “special warnings” about the development of neutropenia and FN [7]. It can be concluded that the introduction of these so-called novel agents did not reduce the incidence of neutropenia because of the strong intrinsic neutropenic effect of IMiD (lenalidomide and pomalidomide) and also because other novel agents without a significant innate neutropenic innate effect (such as monoclonal antibodies or PI) are often used in combination with IMiD.

## Pharmacology of pegfilgrastim

The recombinant methionyl human G-CSF filgrastim, introduced into clinical practice in 1991, works mainly by inducing the proliferation and differentiation of committed progenitor cells of the granulopoietic lineage into functionally mature neutrophils [37]. When administered subcutaneously, filgrastim can induce a rapid increase in the ANC within 24 h, requiring continuous daily injections to reach an adequate ANC level (about 5 days) [38]. Filgrastim pharmacokinetics, in particular its clearance, is inversely related to the ANC. The mean half-life is 4.7 h at an ANC of 0 but < 2 h when the ANC is > 17.0 × 10(9)/L [39]. These data suggest a complex physiological model of filgrastim clearance, with two main pathways. Like other cytokines and growth factors (thrombopoietin, erytropoietin, etc.) whose production is inhibited by an increase in the cells that are biologically targeted by them in a negative feedback manner, G-CSF synthesis is reduced when the ANC increases, as shown in preclinical and clinical models [40–43]. There is in vitro evidence that a G-CSF clearance process is directly mediated by a specific receptor expressed on the surface of mature neutrophils [44]. In addition to the mechanism directly involving neutrophils, G-CSF removal is also governed by renal excretion [45, 46]. Thus, there are two main pathways that determine G-CSF removal and half-life shortening: renal function, capable of constant renal clearance even with a very low ANC, and a modulatory (and theoretically saturable) pathway mediated by the number of circulating neutrophils. The consequences of this complex clearance mechanism are that patients require subsequent daily subcutaneous injections of filgrastim to obtain an adequate ANC level and that there is no universally standardized schedule of administration (may be administered either every day or every other day) [47]. The introduction of the pegylated form, pegfilgrastim, has overcome this clinical issue thanks to covalent conjugation of proteins with poly-ethylene–glycol (PEG), a standard pharmaceutical strategy used to prolong half-life and improve the clinical benefit of different kind of molecules with biological activity (e.g., peg-interferon, peg-asparaginase) [48–50]. Pegfilgrastim has the same biological properties as filgrastim, including the stimulation of the proliferation and differentiation of neutrophil precursors into mature cells. It also has the advantage of a longer-lasting half-life, leading to a single administration per cycle thanks to diminished renal clearance because of the reduced permeability of glomerular barrier by a larger molecule [48, 51, 52].

## Pegfilgrastim as primary prophylaxis in multiple myeloma

Pegfilgrastim is an effective mobilizing agent during ASCT for different hematological neoplasms, including patients with MM undergoing this procedure, but its role in this setting is outside the scope of the present review [53–55]. It has also become widely used in Europe thanks to the fact that it can be administered in a single subcutaneous injection at a fixed time (at least 24 h after therapy) and at a fixed dose (6 mg), which can substantially improve patient compliance to receive this fundamental supportive measure. Moreover, although no direct comparisons have been made, there is evidence to suggest that pegfilgrastim is superior to filgrastim in reducing neutropenia and FN [56, 57]. Like filgrastim, there are two alternative strategies for its use in MM, i.e. as a primary and secondary prophylaxis [58]. The latter or “*on demand*” prophylaxis is only recommended for treatment regimens with low rates of neutropenia and FN and is based on multiple injections of filgrastim when severe neutropenia or neutropenia-related symptoms appear [59]. For many clinicians, this strategy may seem inadequate to protect frail patients, as MM patients often are, from the risk of severe infection. Pegfilgrastim-based primary prophylaxis is highly recommended during regimens at high risk of causing severe neutropenia (expected incidence > 20%), i.e., lenalidomide-containing regimens or pomalidomide-containing regimens [58]. This strategy is also highly recommended for patients undergoing treatments with an intermediate low risk of neutropenia (e.g., bortezomib-based triple-drug regimens) and for those harboring risk factors for neutropenia (e.g., age > 65 years, comorbidities, low performance status, poor nutritional status) [58]. Primary prophylaxis is also recommended for patients with an expected rate of FN > 20% [59]. The use of primary prophylaxis in MM starting from the first courses of treatment reduces the risk of severe infections, which is highest in the first 2 months, and decreases the need for premature dose adjustment or treatment discontinuation, conditions that could potentially impede an initial and rapid breakdown of disease burden. Primary prophylaxis with pegfilgrastim has been investigated less frequently than the use of the G-CSF in ASCT. Three trials investigating a lenalidomide-based regimen, i.e., regimens harboring a high risk of neutropenia and infections, used pegfilgrastim for primary prophylaxis. The German Myeloma Study Group (DSMM) conducted a phase 1–2 trial exploring the efficacy and tolerability of the lenalidomide, adriamycin, and dexamethasone combination in rrMM, delivered as a 28-day cycle [60]. Pegfilgrastim 6 mg was administered on the 6th day of each cycle, and after four cycles, the dose level of the drugs was evaluated without the use of G-CSF support. Primary support with pegfilgrastim enabled the maximum tolerated dose of lenalidomide (25 mg) to be reached, with neutropenia and infection rates of 48% and 10.5%, respectively (grades 3 and 4). The possibility of increasing the maximum tolerated dose level of lenalidomide resulted in in a better response, in terms of ORR [60]. Neutropenia (grade 3–4) was also explored in a prospective observational study in which rrMM patients underwent treatment with lenalidomide-dexamethasone [61]. Neutropenia and febrile neutropenia rates were 31% and 3%, respectively, but the significance of primary prophylaxis with pegfilgrastim was weak as this specific G-CSF support modality was only used in 8% of patients. A prospective, multicenter phase 2 trial was conducted to assess the efficacy and safety of the bendamustine-lenalidomide-dexamethasone combination in rrMM [62]. Pegfilgrastim was administered to 68% of patients as primary prophylaxis at a dose of 6 mg on day 3 of each 28-day cycle. In this regimen, where another strong neutropenia-inducing drug such as bendamustine was added to lenalidomide, the high rate of grade 3–4 neutropenia (74%) and other hematological toxicities was concerning, and pegfilgrastim supportive care was fundamental to ensuring the adequate delivery of the planned treatment. These are not data from a study whose main objective was to assess primary prophylaxis with pegfilgrastim, but rather are ancillary information obtained from the overall data on safety. More insights into pegfilgrasim prophylaxis can also be obtained from real-life surveys. In an observational study, 41 patients with heavily pre-treated rrMM exposed to multiple types of anti-myeloma agents underwent primary prophylaxis with pegfilgrastim 6 mg on day 3 from the second course of treatment onwards after a first cycle in which multiple injections of filgrastim were administered [63]. Pegfilgrastim proved superior to filgrastim in terms of both quality of response to neutropenia (evaluating absolute neutrophils at nadir) and duration of neutropenia, which was shorter in the cycles supported by pegfilgrastim [63]. This led to a reduction in the rate of neutropenia and infection and enabled the established treatment schedule to be delivered to a cohort of very frail patients. Similarly, another cohort of 47 rrMM patients given bendamustine-bortezomib-dexamethasone as salvage therapy was studied to evaluate the superior efficacy of pegfilgrastim prophylaxis over filgrastim phophylaxis in this regimen with its potentially high risk of neutropenia [64]. Twenty-four patients underwent pegfilgrastim 6 mg on day 4, while the remaining 23 received “*on demand*” filgrastim when ANC dropped < 1000 cells/mL. Prophylaxis with pegfilgrastim was significantly associated with lower FN-related chemotherapy disruption rates (8.3 vs 17.3% in the pegfilgrastim and filgrastim groups, respectively) and fewer FN-related days in hospital (0 vs 15 days, respectively). There was also a decrease in the rate of infection that enabled the scheduled chemotherapy to be administered, without the need for (lengthy) treatment discontinuation [65]. In these clinical experiences, pegfilgrastim is tolerated as well as if not better than filgrastim, with the classic adverse events associated with G-CSF (mild fever and bone pain, easily manageable with paracetamol) [63, 64]. Table 1 provides an overview of the reported studies.

**Table 1:** An overview of the reported studies

References	Study design	Patients and treatment	PEG schedule	Results	Comments
Knop et al. [60]	Phase1/trial, MTD evaluation	190 RRMM patients undergoing RAD	Pegfilgrastim 6 mg s.c. day + 6 as primary prophylaxis	Grade 3–4 neutropenia and infections rates were 48% and 10.5%, respectively	PEG prophylaxis led to better tolerability of lenalidomide-related neutropenia, thus providing better outcomes
Leleu et al. [61]	Prospective, observational	198 MM patients undergoing lenalidomide plus dexamethasone	Only 16 patients on primary prophylaxis with PEG (no dose reported)	12 patients had a reduction of lenalidomide exposure and long GCSF course	Some patients would have been well tolerated lenalidomide with PEG prophylaxis instead of GCSF support
Mey et al. [62]	Prospective phase 2	50 RRMM patients undergoing bendamustine-lenalidomide-dexamethasone	Pegfilgrastim 6 mg s.c. day + 3 as primary prophylaxis	Grade 4 neutropenia 34% of patients	PEG prophylaxis limited bendamustine toxicity, leading to better outcome
Cerchione et al. [63]	Observational	41 heavily pre-treated RRMM patients supported with GCSF or PEG	Pegfilgrastim 6 mg s.c. day + 3	Neutropenia duration was shorter and quantitatively reduced in PEG primary prophylaxis setting vs GCSF on demand setting	PEG provided better tolerability of treatment regimens, with less treatment disruption neutropenia or FN-related
Cerchione et al. [64]	Observational	47 heavily pre-treated RRMM patients supported with GCSF or PEG while on bendamustine	Pegfilgrastim 6 mg s.c. day + 4	Neutropenia duration was shorter and quantitatively reduced in PEG primary prophylaxis setting vs GCSF on demand setting	PEG provided better tolerability of treatment regimens, with less treatment disruption neutropenia or FN-related

## Conclusions

Primary prophylaxis with pegfilgrastim in MM is feasible, safe, and effective. Many clinical data, although mostly observational, address the superiority of pegfilgrastim prophylaxis over filgrastim, especially in neutropenia high-risk treatment [65, 66]. However, the lack of prospective trials in this field has prevented guidelines from being established for clinicians. The choice between the two delivery modalities of G-CSF support, i.e., primary prophylaxis or secondary prophylaxis, is still based on clinical and non-clinical factors. There are several factors pointing to the advantage of primary prophylaxis with pegfilgrastim over “on demand” filgrastim: (1) the fact of having one fixed administration rather than multiple injections, which positively impacts quality of life; (2) the possibility of a more effective prevention of infections, the main cause of death in MM patients; and (3) the reduction in overall costs (days in hospital, use of anti-infective drugs, and other diagnostic and therapeutic measures for severe infection) for patients receiving supportive care with pegfilgrastim. Overall, expert opinion on indication of primary prophylaxis in multiple myeloma recommends the adoption of this strategy in case of high-risk neutropenia treatments, such as triplets lenalidomide-containing regimens, or in low/intermediate-risk treatments plus other risk factors [59]. Actually, these indications on pegfilgrastim use are still valid, but probably, when feasible, should be also extended to majority of patients due to the clinical and cost-effectiveness advantages abovementioned. Still, probably, new observational or prospective trials are needed in view of the rapid development and spread of everyday clinical practice of the use of plenty of new anti-myeloma agents that deserves adequate supportive strategy when combined with lenalidomide or, perhaps, as single agents such as pomalidomide or daratumumab.
